# A comparative analysis of different biofluids towards ovarian cancer diagnosis using Raman microspectroscopy

**DOI:** 10.1007/s00216-020-03045-1

**Published:** 2020-11-26

**Authors:** Panagiotis Giamougiannis, Camilo L. M. Morais, Rita Grabowska, Katherine M. Ashton, Nicholas J. Wood, Pierre L. Martin-Hirsch, Francis L. Martin

**Affiliations:** 1 Department of Obstetrics and Gynaecology, Lancashire Teaching Hospitals NHS Foundation Trust, Preston, PR2 9HT UK; 2 School of Pharmacy and Biomedical Sciences, University of Central Lancashire, Preston, PR1 2HE UK; 3 Department of Pathology, Lancashire Teaching Hospitals NHS Foundation Trust, Preston, PR2 9HT UK; 4 Biocel Ltd, Hull, HU10 7TS UK

**Keywords:** Ovarian cancer, Biofluids, Liquid biopsies, Raman spectroscopy, Spectroscopy

## Abstract

Biofluids, such as blood plasma or serum, are currently being evaluated for cancer detection using vibrational spectroscopy. These fluids contain information of key biomolecules, such as proteins, lipids, carbohydrates and nucleic acids, that comprise spectrochemical patterns to differentiate samples. Raman is a water-free and practically non-destructive vibrational spectroscopy technique, capable of recording spectrochemical fingerprints of biofluids with minimum or no sample preparation. Herein, we compare the performance of these two common biofluids (blood plasma and serum) together with ascitic fluid, towards ovarian cancer detection using Raman microspectroscopy. Samples from thirty-eight patients were analysed (*n* = 18 ovarian cancer patients, *n* = 20 benign controls) through different spectral pre-processing and discriminant analysis techniques. Ascitic fluid provided the best class separation in both unsupervised and supervised discrimination approaches, where classification accuracies, sensitivities and specificities above 80% were obtained, in comparison to 60–73% with plasma or serum. Ascitic fluid appears to be rich in collagen information responsible for distinguishing ovarian cancer samples, where collagen-signalling bands at 1004 cm^−1^ (phenylalanine), 1334 cm^−1^ (CH_3_CH_2_ wagging vibration), 1448 cm^−1^ (CH_2_ deformation) and 1657 cm^−1^ (Amide I) exhibited high statistical significance for class differentiation (*P* < 0.001). The efficacy of vibrational spectroscopy, in particular Raman spectroscopy, combined with ascitic fluid analysis, suggests a potential diagnostic method for ovarian cancer.

**Figure:**
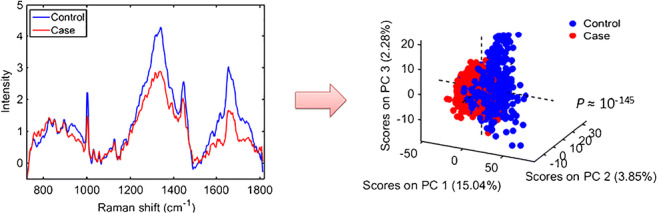
Raman microspectroscopy analysis of ascitic fluid allows for discrimination of patients with benign gynaecological conditions or ovarian cancer.

Raman microspectroscopy analysis of ascitic fluid allows for discrimination of patients with benign gynaecological conditions or ovarian cancer.

## Introduction

Ovarian cancer is the seventh most commonly occurring cancer in women worldwide, with nearly 300,000 new cases diagnosed in 2018 [1]. In the UK, it is the sixth most common cancer in women, with around 7400 new diagnoses every year. It is also a leading cause of mortality from gynaecological malignancies, accounting for 5% of all cancer deaths in females [2]. This is because its presentation is notoriously non-specific with symptoms that are widely experienced among the general population; hence, most women tend to present with advanced disease [3]. Therefore, its early detection represents the best hope for mortality reduction.

A common feature of women with ovarian cancer is the presence of ascites (accumulation of free fluid in the peritoneal cavity). Normally, capillary membranes continuously produce free fluid to keep the serosal surfaces of the peritoneal lining lubricated, with two-thirds being reabsorbed into lymphatic channels [4]. In cases of disseminated intra-abdominal cancer (such as ovarian cancer), exaggerated production of peritoneal fluid is induced due to increased leakiness of tumour microvasculature and obstruction of lymphatic vessels [5]. More than one-third of ovarian cancer patients present with significant ascites at diagnosis [6].

Currently used diagnostic modalities include clinical examination, imaging (such as ultrasound and computed tomography), measurement of serum cancer antigen CA125 and tissue biopsies. However, the aforementioned tools are often invasive, expensive and time-consuming. These limitations fuel the need for development of new less-invasive, quicker, lower-cost and sensitive methodologies for ovarian cancer detection. One of these promising new methodologies is vibrational spectroscopy.

Vibrational spectroscopy is a bio-analytical tool that has the potential to classify normal and pathological tissue [7]. Several spectroscopic techniques have been utilised in the past decades to detect structural alterations that occur in molecules within cells, according to their chemical bonds [8]. Biomolecules undergo vibrational changes following irradiation with infrared (IR), which can be detected within the IR region of the electromagnetic spectrum as discrete wavenumber absorption intensities, or following inelastic light scattering of a monochromatic laser source (Raman effect), where Raman shifts in the wavenumber frequency, representing molecular polarisability changes, are detected [9].

Raman is a powerful technique for biological materials analysis, with applications ranging from cell imaging to cancer diagnosis [10–12]. Regarding ovarian cancer, Raman spectroscopy of tissue [13, 14] as well as blood plasma and serum [15–18] has been reported. The main advantages of using biofluids rely in that sample preparation is not needed; hence, no reagents are required, and the liquid biopsies processing is less invasive than traditional tissue ones. In addition, spectral profile alterations of biofluids can be determined, making the method suitable for automation [19].

Herein, the potential of Raman microspectroscopy for ovarian cancer detection is explored by comparing the performance of common available biofluids (plasma and serum), together with ascitic fluid spectroscopy. The spectral profiles of ovarian cancer patients are discriminated from patients with benign gynaecological conditions based on a series of computer-aided techniques, such as principal component analysis (PCA), discriminant analysis (DA) and support vector machines (SVM), and each biofluid performance is compared.

## Materials and methods

### Patients and samples

Blood plasma and serum, as well as whole ascites from thirty-eight patients (*n* = 18 with ovarian cancer; *n* = 20 with benign gynaecological conditions—controls), were used for the purposes of this study. The age of the patient cohort, presented as median value (interquartile range), was 61 (54, 73) years and 52 (41, 58) years for the ovarian cancer and benign groups, respectively. Informed consent was taken from all participants. Samples were collected upon patients’ attendance for surgery at the Royal Preston Hospital and therefore were fasting samples. Each pair of blood samples was collected pre-operatively in 7.5-ml tubes containing EDTA anticoagulant and 7.5-ml serum gel tubes. Ascites was collected intra-operatively in 20-ml universal container tubes. All biofluids were initially stored in a fridge at 4–7 °C for up to 2 h. Prior to freezing, blood samples were centrifuged at 20 °C and 2200 rpm for 15 min, to obtain plasma and serum samples (local protocol). Blood plasma was obtained from EDTA tubes and serum (which has the same consistency with plasma but does not contain clotting factors) from serum gel tubes. Ascites was not centrifuged. All biofluids were snap frozen in liquid nitrogen and stored at − 80 °C.

Prior to slide preparation samples were thawed at room temperature. Thirty microliters of individual biofluids (plasma, serum and ascites) were pipetted onto aluminium foil–lined FisherBrand™ glass slides for Raman spectroscopy analysis [20]. Each slide was labelled with a specific GU (genito-urinary) number for patient confidentiality. Samples were left to dry overnight before transportation to the spectroscopy laboratory in the University of Central Lancashire (UCLan) for Raman analysis. All slides were stored in a de-humidified glass container to prevent sample condensation and physical damage. Ethical approval for samples collection was granted by the East of England - Cambridge Central Research Ethics Committee (archival genito-urinary tissue, blood, urine, saliva and ascitic fluid collection; REC reference: 16/EE/0010; IRAS project ID: 195311). Ethical approval for experimental analysis was granted from UCLan (STEMH 1073 application).

Identification of pathology for all recruited patients, as well as staging and grading for ovarian cancer patients, was based on histopathology reports after processing of surgical specimens. Staging of ovarian cancer was conducted according to the International Federation of Gynecology and Obstetrics (FIGO) system [21]. Histology diagnoses of benign controls were (number of cases in brackets) as follows: ovarian cysts (7), leiomyomas +/− adenomyosis (7), endometriosis (4), no pathology identified (normal) (2). Ovarian cysts are fluid-filled sacs on the ovary and leiomyomas (or fibroids) are benign growths in the wall of the uterus. In adenomyosis, the inner lining of the uterus (endometrium) breaks through its muscular layer and in endometriosis tissue similar to the endometrium grows in extrauterine locations [22, 23]. All ovarian cancer patients had epithelial tumours (i.e. originating from the ovarian surface epithelium (OSE)) and relevant histologies were as follows: serous adenocarcinoma (13), clear cell adenocarcinoma (2), endometrioid adenocarcinoma (1), mucinous adenocarcinoma (1), carcinosarcoma (1). Different types represent aberrant differentiation to various non-OSE histologies (fallopian tube-like for serous, endometrium-like for endometrioid and clear cell, endocervical-like for mucinous and containing both epithelial and mesenchymal ovarian components for carcinosarcomas) [24, 25]. With regard to FIGO staging, six patients had stage I disease, one patient stage II, nine patients stage III and two patients stage IV. None of the ovarian cancer patients had received chemotherapy prior to their surgery. All demographic data (including BMI, comorbidities, medications) are available in non-patient identifiable databases.

### Spectral acquisition

A Renishaw InVia Basis Raman spectrometer coupled to a confocal microscope (Renishaw plc, UK) was used for spectral acquisition. Samples were analysed with an acquisition area of approx. 250 × 125 μm using × 20 and laser power of 10% at 785 nm with 0.1-ms exposure time. The exposure time was kept reduced to avoid sample damage. Spectral mapping was acquired via StreamHR™ technique (high-confocality mode) with a grid area of approx. 23 × 15 pixels, resulting in 345 spectra per sample in an acquisition time of approximately 10 min per sample. The spectral range was set between 725 and 1813 cm^−1^ with 1 cm^−1^ spectral resolution.

### Computational analysis

Raman data were converted into suitable files using the Renishaw WiRE software and processed using MATLAB R2014b (MathWorks, Inc., USA). Raman mapping data for each sample were firstly unfolded into two-dimensional matrices (*n* rows—spectra, *m* columns—wavenumbers) and averaged every 10 spectra to reduce data size and speed up the computational analysis time. Resultant data were pre-treated by spike (cosmic rays) removal.

Three combinations of spectral pre-processing techniques were tested for data analysis: (i) Savitzky-Golay (SG) smoothing (window of 7 points, 1^st^-order polynomial fitting) followed by automatic weighted least squares (AWLS) baseline correction (3^rd^-order fit) and vector normalisation; (ii) SG smoothing (window of 7 points, 1^st^-order polynomial fitting) followed by extended multiplicative scatter correction (EMSC) and AWLS baseline correction (3^rd^-order fit) and (iii) SG smoothing (window of 7 points, 1^st^-order polynomial fitting) and 1^st ^derivative. SG smoothing corrects for random noise, AWLS baseline correction and 1st derivative correct for baseline distortions, and EMSC and vector normalisation correct for physical differences between samples such as thickness, light scattering and concentrations (for vector normalisation) [26]. Exploratory and discriminant analyses were performed with the pre-processed and mean-centred data.

Principal component analysis (PCA) [27] was used for exploratory analysis. PCA reduces the pre-processed spectral dataset into a small number of principal components (PCs), responsible for the majority of data variance. Each PC is composed of scores and loadings; the former is used to access similarity/dissimilarity patterns among samples and the latter to identify spectral features (wavenumbers), associated with class separation and therefore possible spectral biomarkers. For discrimination, the PCA score data were further analysed by linear discriminant analysis (PCA-LDA), quadratic discriminant analysis (PCA-QDA) and support vector machines (PCA-SVM). PCA models were built using the PLS Toolbox version 7.9.3 (Eigenvector Research, Inc., USA) and discriminant analysis was performed using the Classification Toolbox for MATLAB [28]. Furthermore, partial least squares discriminant analysis (PLS-DA) [29] was also used as a comparative technique.

LDA and QDA are based on the calculation of the Mahalanobis distance between samples [30]. The main difference between them is that LDA considers the classes (i.e. benign controls and ovarian cancer) having similar variance structures, thus building a model based on a pooled covariance matrix, whereas QDA considers the classes having different variance structures, thus building a model based on an independent variance-covariance matrix for each class individually. On the other hand, SVM is a more complex machine learning technique that classifies samples by using a non-linear step called the kernel transformation [31]. The kernel function, which herein was the radial basis function (RBF), non-linearly projects data into a feature dimension, where samples are classified based on a linear threshold. The RBF kernel is able to adjust to different data distributions and SVM tends to be a more powerful discriminant technique, though more susceptible to over-fitting [26]. PLS-DA is one of the most popular supervised classification techniques, based on a linear model for which the classification criterion is obtained by PLS [32]. In PLS-DA, PLS is applied to data reducing the original variables (wavenumbers) to a few numbers of latent variables in an iterative process, where the class labels for each sample are known in the training set. Then, a straight line that divides the classes’ regions is found [20, 29].

### Statistical analysis

The discriminant models were evaluated by calculating some metrics (accuracy, sensitivity, specificity and *F*-score) via 10-fold cross-validation. Accuracy represents the proportion of true positives and true negatives in all evaluated cases. Sensitivity and specificity represent the proportions of positives (i.e. ovarian cancers spectra) and negatives (i.e. benign controls spectra) correctly identified, respectively. The *F*-score measures the overall model performance considering imbalanced data [33]. These parameters are calculated as follows:

1 $$ \mathrm{Accuracy}\ \left(\%\right)=\left[\left(\mathrm{TP}+\mathrm{TN}\right)/\left(\mathrm{TP}+\mathrm{FP}+\mathrm{TN}+\mathrm{FN}\right)\right]\times 100 $$

2 $$ \mathrm{Sensitivity}\ \left(\%\right)=\left[\mathrm{TP}/\left(\mathrm{TP}+\mathrm{FN}\right)\right]\times 100 $$

3 $$ \mathrm{Specificity}\ \left(\%\right)=\left[\mathrm{TN}/\left(\mathrm{TN}+\mathrm{FP}\right)\right]\times 100 $$

4 $$ F-\mathrm{score}\ \left(\%\right)=\left(2\times \mathrm{SENS}\times \mathrm{SPEC}\right)/\left(\mathrm{SENS}+\mathrm{SPEC}\right) $$

where TP stands for true positives, TN for true negatives, FP for false positives and FN for false negatives. SENS stands for sensitivity and SPEC for specificity. *P*-values were calculated for three-dimensional PCA score plots using a MANOVA test and for individual wavenumbers based on an ANOVA test. Statistical significance was considered at *P* < 0.05 and statistical high significance at *P* < 0.001.

## Results

**Fig. 1:**
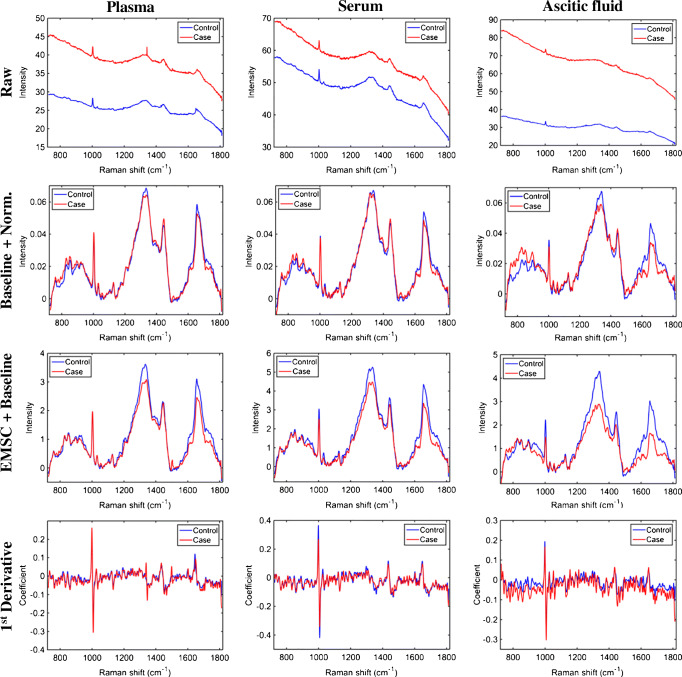
Average raw and pre-processed Raman spectra for controls (patients with benign gynaecologic conditions) and cases (ovarian cancer patients) for different biofluids and pre-processing techniques: ‘Raw’ for raw data; ‘Baseline + Norm.’ for SG smoothing followed by AWLS baseline correction and vector normalisation; ‘EMSC + Baseline’ for SG smoothing followed by EMSC and AWLS baseline correction; and ‘1^st ^Derivative’ for SG smoothing followed by 1^st^ derivative

The raw and pre-processed spectra for cases (ovarian cancers) and controls (patients with benign gynaecological conditions) are shown in Fig. 1. Three different pre-processing approaches were applied to the raw dataset: the first (Baseline + Norm.) being SG smoothing followed by AWLS baseline correction and vector normalisation, which removes random noise, corrects the baseline and different sample thickness and concentrations; the second (EMSC + Baseline) was SG smoothing followed by EMSC and AWLS baseline correction, which has an effect similar to the previous pre-processing, but it corrects for light scattering and does not correct for samples with different concentrations (one of the effects of vector normalisation); and the third approach (1st derivative) was SG smoothing followed by 1st derivative, which corrects for random noise, baseline, sample thickness and light scattering, but increases substantially the noise level [26].

**Fig. 2:**
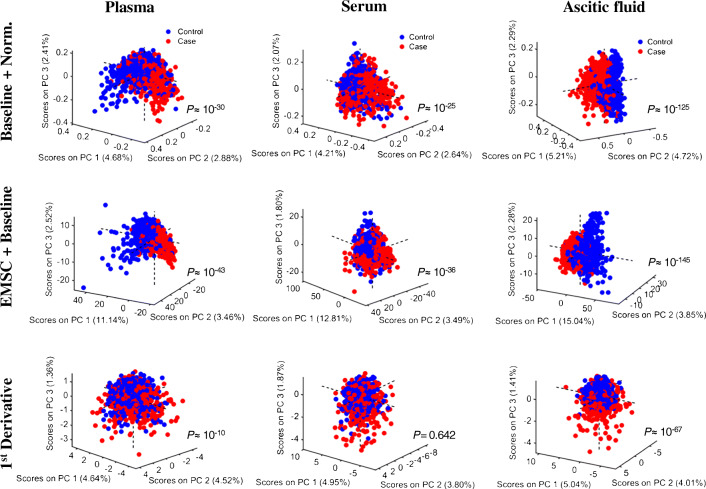
Three-dimensional PCA score plot (PC1 vs. PC2 vs. PC3) for different biofluids and pre-processing techniques: ‘Baseline + Norm.’ for SG smoothing followed by AWLS baseline correction and vector normalisation; ‘EMSC + Baseline’ for SG smoothing followed by EMSC and AWLS baseline correction; and ‘1^st^ Derivative’ for SG smoothing followed by 1^st^ derivative. Numbers inside parentheses represent the variance on each PC direction. *P*-values calculated based on a MANOVA test

Exploratory analysis was performed with PCA. The PCA score plots for the three types of pre-processed data are shown in Fig. 2, where *P-*values were calculated based on the three-dimensional score plot by using a MANOVA test with the score values on PC1, PC2 and PC3. Visually and based on *P-*values, it is evident that the second pre-processing approach (EMSC + Baseline) had the greatest potential for discrimination. It exhibited the clearest clustering separation in all three types of biofluids and the lowest *P-*values in plasma (*P* ≈ 10^−43^), serum (*P* ≈ 10^−36^) and ascitic fluid (*P* ≈ 10^−145^). First derivative had the highest *P-*values and did not show statistical significance in serum (*P* = 0.642). Among biofluids, ascitic fluid had the lowest *P*-values, followed by plasma and then serum.

Following exploratory analysis, discriminant analysis algorithms were applied to the PCA scores (PCA-LDA, PCA-QDA and PCA-SVM) and to the original pre-processed data by means of PLS-DA. Classification was performed on a spectral basis to reduce the risk of over-fitting in SVM. However, SVM-based models carry a higher risk of over-fitting due to the small sample cohort used in this study. Discriminant analysis results are shown in Table 1 where accuracy, sensitivity, specificity and *F*-score are reported for each model. EMSC + Baseline pre-processing achieved the best discrimination among biofluids and ascites had the best discrimination performance overall. The combination of EMSC + Baseline pre-processing and ascitic fluid generated the best discriminant analysis model, with an overall performance of *F*-score = 82% both for PCA-LDA (sensitivity = 84%, specificity = 81%) and PCA-SVM (sensitivity = 78%, specificity = 86%), which were the best discriminant algorithms. In this case, discriminant results were similar among the different types of algorithm, with PCA-SVM > PLS-DA > PCA-QDA. Between PCA-LDA and PCA-SVM, the former has more well-balanced sensitivities and specificities, is the simplest method (more parsimonic), is more robust and is less susceptible to over-fitting [26, 34], hence being the method of choice.

**Table 1:** Discriminant analysis results for plasma, serum and ascitic fluid with different pre-processing techniques: ‘Baseline + normalisation’ for SG smoothing followed by AWLS baseline correction and vector normalisation; ‘EMSC + baseline’ for SG smoothing followed by EMSC and AWLS baseline correction; and ‘1^st ^Derivative’ for SG smoothing followed by 1^st^ derivative. PCs, principal components; LVs, latent variables

		**Accuracy (%)**	**Sensitivity (%)**	**Specificity (%)**	***F*-score (%)**
**Plasma**	**Baseline + normalisation**				
	PCA-LDA (7 PCs)	65	56	73	63
	PCA-QDA (5 PCs)	65	49	79	60
	PCA-SVM (7 PCs)	68	56	78	65
	PLS-DA (2 LVs)	65	57	73	64
	**EMSC + baseline**				
	PCA-LDA (6 PCs)	66	60	72	65
	PCA-QDA (3 PCs)	63	78	48	59
	PCA-SVM (6 PCs)	67	65	69	67
	PLS-DA (2 LVs)	66	69	63	66
	**1st Derivative**				
	PCA-LDA (8 PCs)	58	41	73	53
	PCA-QDA (8 PCs)	59	29	86	43
	PCA-SVM (8 PCs)	59	48	70	57
	PLS-DA (8 LVs)	61	52	68	59
**Serum**	**Baseline + normalisation**				
	PCA-LDA (6 PCs)	65	56	72	63
	PCA-QDA (5 PCs)	70	57	82	67
	PCA-SVM (5 PCs)	68	57	77	66
	PLS-DA (3 LVs)	64	59	68	63
	**EMSC + baseline**				
	PCA-LDA (4 PCs)	66	59	72	65
	PCA-QDA (4 PCs)	67	71	63	67
	PCA-SVM (4 PCs)	73	61	84	71
	PLS-DA (3 LVs)	65	67	64	65
	**1st Derivative**				
	PCA-LDA (6 PCs)	65	56	72	63
	PCA-QDA (5 PCs)	70	57	82	67
	PCA-SVM (5 PCs)	71	59	81	68
	PLS-DA (3 LVs)	64	59	68	63
**Ascitic fluid**	**Baseline + normalisation**				
	PCA-LDA (2 PCs)	77	71	83	77
	PCA-QDA (3 PCs)	78	75	80	77
	PCA-SVM (3 PCs)	81	82	80	81
	PLS-DA (2 LVs)	79	76	81	78
	**EMSC + baseline**				
	PCA-LDA (6 PCs)	82	84	81	82
	PCA-QDA (2 PCs)	80	86	75	80
	PCA-SVM (6 PCs)	82	78	86	82
	PLS-DA (2 LVs)	81	87	76	81
	**1st Derivative**				
	PCA-LDA (4 PCs)	72	60	83	70
	PCA-QDA (3 PCs)	71	54	86	66
	PCA-SVM (4 PCs)	72	57	85	68
	PLS-DA	76	64	87	74

**Fig. 3:**
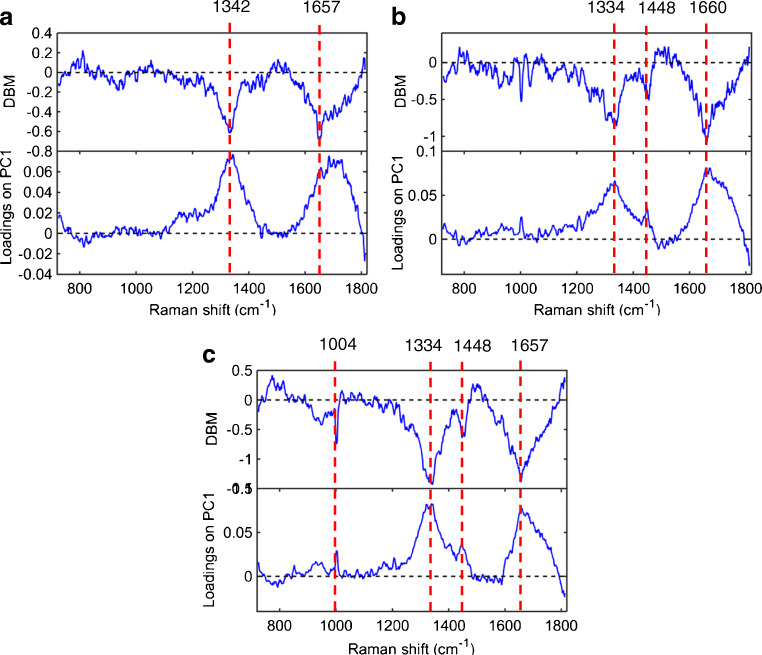
Difference-between-mean (DBM) spectrum and PCA loadings on PC1 (for the pre-processed data using SG smoothing followed by EMSC and AWLS baseline correction) for (**a**) plasma, (**b**) serum and (**c**) ascitic fluid. Raman shifts (in cm^−1^) are assigned for the main bands

The wavenumbers responsible for class differentiation were identified by combining the difference-between-mean (DBM) spectrum with the PCA loadings on PC1 (main discriminant direction). The DBM is produced following subtraction between the mean spectrums of ovarian cancer and benign control classes, using the best pre-processing approach (EMSC + Baseline). This spectrum indicates the main differences between the two classes. PCA loadings give more refined details about class differentiation, as they show wavenumbers with the highest weights towards discrimination on the PC direction. The coincidence in these two points of information indicates that the wavenumbers on them are greatly significant for class separation [35] (Fig. 3). Regarding ascitic fluid, four wavenumbers (1004, 1334, 1448 and 1657 cm^−1^) were mainly responsible for class differentiation (Fig. 3c), while for plasma and serum, two and three wavenumbers were identified, respectively (Fig. 3a and b). All wavenumbers identified as discriminant features in plasma, serum and, particularly, ascitic fluid are somehow related to collagen [36], and all of them are less intense in the case (ovarian cancers) class (Table 2).

**Table 2:** Tentative spectral biomarker assignment [36]. ↓ stands for lower Raman intensity. *P* values calculated based on an ANOVA test

Biofluid	Wavenumber (cm^−1^)	Tentative assignment	Intensity in ‘Case’	*P* value
Plasma	1342	CH deformation (proteins and carbohydrates)	↓	10^−17^
	1657	Amide I (collagen)	↓	10^−24^
Serum	1334	CH_3_CH_2_ wagging (collagen)	↓	10^−14^
	1448	CH_2_ deformation (collagen)	↓	10^−06^
	1660	Amide I (proteins)	↓	10^−24^
Ascitic fluid	1004	Phenylalanine (collagen)	↓	10^−22^
	1334	CH_3_CH_2_ wagging (collagen)	↓	10^−56^
	1448	CH_2_ deformation (collagen)	↓	10^−19^
	1657	Amide I (collagen)	↓	10^−62^

## Discussion

Clinical spectroscopy comprises powerful tools used to obtain spectrochemical information of biological materials. The spectral biofingeprint around 1800 to 900 cm^−1^ generated by these techniques contains vibrational information of key biomolecules (such as proteins, lipids, carbohydrates and nucleic acids), thus being used as a numeric source of data for disease diagnosis [8]. Herein, Raman microspectroscopy was used to identify the potential of plasma, serum and ascites for ovarian cancer detection. Different pre-processing and discriminant analysis approaches were tested, where SG smoothing followed by EMSC and AWLS baseline correction in combination with PCA-LDA was found to be the best method. An initial exploratory analysis (Fig. 2) already suggested that ascitic fluid was the best for differentiation of ovarian cancer from benign gynaecological conditions, where higher statistically significant values (*P* < 0.001) were found in the three-dimensional PCA score plot. Discrimination-wise, PCA-LDA in ascitic fluid was able to separate the classes with accuracies, sensitivities and specificities above 80%, in comparison with 60–73% for plasma and serum (Table 1).

**Fig. 4:**
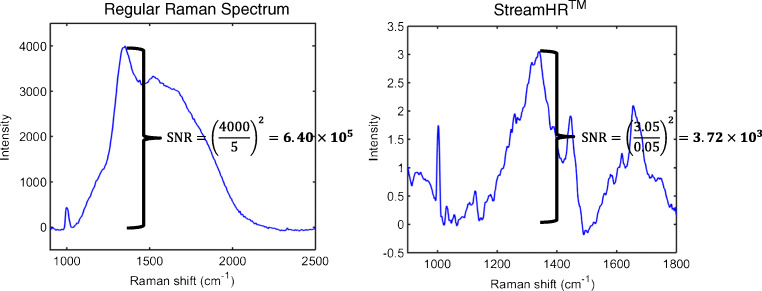
Comparison between the signal-to-noise ratio (SNR) for a regular Raman spectrum and a Raman spectrum acquired by the StreamHR™ technique for ascites

To our knowledge, Raman spectroscopy of ascites is undertaken for the first time. Three studies have conducted Raman spectroscopy of blood-derived biofluids in ovarian cancer, all involving equal numbers of cases and benign controls in their cohorts. The biggest one by Paraskevaidi et al. (27 ovarian cancer patients) found blood plasma to have sensitivity and specificity ranging from 78 to 99% and from 85 to 99%, respectively, using SVM, and ranging from 72 to 96% and from 74 to 97%, respectively, using SVM with surface-enhanced Raman spectroscopy (SERS) [16]. In the smallest study by Owens et al. (2 ovarian cancer patients), a classification accuracy of 74% for plasma was reported, using SVM [17]. Finally in the study by Ullah et al. (11 ovarian cancer patients), spectroscopy of serum yielded 90% sensitivity and 100% specificity, again with SVM [18]. Herein, plasma and serum had lower performances. A possible reason for these differences could be variations in patient characteristics (such as age, BMI, comorbidities) though regression analyses for confounding factors were not conducted in any of the aforementioned studies (including ours). Regarding age, Paraskevaidi et al. performed subgroup analyses in patients who were more or less than 60 years old and found only minor changes in sensitivity and specificity, suggesting that this factor is unlikely to influence outcomes [16]. Another potential reason could be differences in the spectroscopic technique used. The study herein used StreamHR™ to investigate biofluids (i.e. neither single spectral acquisition nor SERS which were used in the three other studies), consequently having a lower signal-to-noise ratio (SNR). Figure 4 shows a SNR comparison between a regular Raman spectrum and a Raman spectrum acquired by StreamHR™ for ascites. The SNR for a regular Raman spectrum is 172 times better than using StreamHR™.

On the other hand, whole ascites processing has advantages compared to blood-derived biofluids. Plasma and serum are obtained following centrifugation of whole blood. However, there are various centrifugation protocols (in terms of centrifugation force and time), which can affect the concentrations of some plasma and serum metabolites through haemolysis [37, 38]. This can increase heterogeneity between studies making comparison of their outcomes less accurate. Additionally, anticoagulants (such as EDTA) and gel contained in the tubes used for plasma and serum collection, respectively, can interfere with analyte concentrations [39]. Ascites can be stored in plain tubes and does not undergo centrifugation, therefore avoiding changes in its constituency. It can be speculated that ascites-derived spectra are more precise in reflecting the true metabolic status of the patient, which might have contributed to the better classification rates they achieved.

More importantly, the use of ascites as a spectroscopy biofluid may have some crucial biological advantages. One of these is that it is far more physiologically targeted, i.e. it consists a more accurate representation of the tumour microenvironment being the exudate of malignant cells; this could reduce patient heterogeneity in large cohorts, where a variety of metabolic factors inherent to each individual would complicate the testing of biofluids taken distant from the site of the lesion. There is evidence that ascites hosts events in ovarian tumourigenesis earlier than in peripheral blood, which could lead to earlier spectroscopic changes [40]. The concentration of inflammatory soluble factors (such as cytokines) is usually much higher in ascites compared to blood, providing an excellent source for the discovery of prognostic biomarkers [41]. Additionally, the increased capillary permeability observed in malignancies favours a shift of protein from the blood vessels into the peritoneal fluid [6]. Diet-related factors, such as malnutrition frequently observed in patients with cancer, can decrease even further the protein concentration in peripheral blood [42]. However, not all ovarian cancer patients suffer from malnutrition and not all malignant ascites have increased protein content compared to benign ones [43]. Therefore, it is questionable whether these factors contribute in better classifications (such as the ones achieved in our study with ascites-derived spectra). They do appear to play a role though and could have partially accounted for the differences observed in blood plasma and serum performance between our study and the aforementioned ones.

Malignant and benign ascites do, however, have some marked differences in their composition, which could result in more pronounced spectral variations between the two classes. A study using nuclear magnetic resonance (^1^H NMR) spectroscopy showed that malignant ascites are much more abundant in lactate and ketone bodies (BHBT, acetoacetate, acetone) and have lower glucose and citrate concentrations compared to benign ones. These differences reflect the increased energy demands of malignant cells, leading to accelerated glucose consumption and consequent initiation of lipolysis, as glucose stores get gradually depleted [43].

The spectral biomarker analysis in Fig. 3 and Table 2 suggests that collagen was the main discriminant factor between cases and benign controls, with lower intensity wavenumbers in ovarian cancer for all biofluids and a wider difference in ascitic fluid. Collagen has an important role in malignancy, where it participates in the processes of cancer cell proliferation, migration and metastasis [44, 45]. Furthermore, in ovarian cancer, there is marked degradation of collagen present in the extracellular matrix under the peritoneal mesothelium during the formation of metastases [46]. In radioimmunoassay studies, ascites and serum concentrations of collagen propeptides and degradation products were higher in ovarian cancer compared to patients with benign ovarian cysts [47, 48]. This is in discrepancy with our finding of lower collagen signal intensity in ovarian cancer, potentially reflecting variable levels of collagen synthesis and proteolysis in malignancy, and would require further investigation. Interestingly, these collagen-related peptides were more abundant (up to 200 times higher) in ascites compared to blood in both malignant and benign states [47, 48]. In addition, concentration differences between cancer and benign patients were wider in ascites for the majority of these peptides [48]. These findings highlight once again the biological advantages of using ascites as an experimental biofluid in ovarian cancer research and may provide another explanation for the better classification rates it achieved in our study compared to blood plasma and serum spectroscopy.

Currently, there are no reliable screening tests for the detection of ovarian cancer. The initial step in women presenting with suspicious symptoms is the performance of a serum CA125 test. However, this test has poor sensitivity and specificity, much lower to the ones achieved with biofluid spectroscopy, as many other gynaecological conditions (such as benign ovarian cysts, endometriosis, uterine fibroids) and non-gynaecological cancers (such as gastrointestinal ones) can raise it. Additionally, only 50% of patients with FIGO stage I ovarian cancer have elevated CA125 levels [49–51]. Predictive models used to differentiate benign from malignant ovarian tumours, such as the Risk of Malignancy Index (RMI) or the International Ovarian Tumour Analysis (IOTA) rules, have improved sensitivities and specificities (ranging between 70 and 93%) [52]. These models use ultrasonography alone or in combination with serum CA125 levels, but rely on the presence of a tumour mass. Ascites can exist in patients lacking a distinct mass and, apart from ovarian cancer, it can have significant volumes in benign gynaecological conditions as well, though less frequently [53]. Nevertheless, in our study, patients were recruited randomly irrespective of presence or absence of ascites in their pre-operative investigations, eliminating selection bias in the spectral performances. On the other hand, although all our patients did have a certain degree of ascites identified intra-operatively and collected for our research, it can be entirely absent in benign gynaecological conditions or even ovarian cancer, which would preclude its universal use for diagnostic purposes through spectroscopy.

Ascitic fluid spectroscopy may, however, have some promising clinical applications. For example, it could provide an alternative to cytological examination of ascites collected during diagnostic procedures, which is limited by very low sensitivity (40–62%) for detection of malignant cells [43]. It could also help to determine the extent of staging laparotomy, when it is doubtful whether an ovarian mass is cancerous. In these cases, intra-operative consultation by a pathologist is pursued, which utilises “frozen section” of the specimen, but has several limitations (such as sampling difficulties and interpretation errors). It also increases the surgical time, with potentially higher patient morbidity, and adds significantly to the cost of the operation [54]. Importantly, it is currently undertaken only in tertiary gynaecology oncology centres and requires histopathologists with expert specialist knowledge, limiting the availability of its use. In contrast, ascitic fluid spectroscopy only takes a few minutes, is much cheaper and does not require specialised medical professionals.

A drawback of ascites is its more complicated acquisition compared to other readily collectable biofluids, such as blood. It requires a surgical procedure (paracentesis), which is undertaken by trained medical specialists, requires the use of anaesthesia (usually local) and has associated uncommon but significant risks (bleeding, infection, injury to intra-abdominal organs) [55]. In contrast, blood collection is performed through simple phlebotomy which is minimally invasive, has minor risks (such as bruising at the puncture site) and can be conducted by non-medical healthcare professionals. Another issue is the timely identification of ascitic fluid. There is a progressive relationship between ovarian cancer stage and proportion of cases with ascites as well as quantity of ascites. As patients can remain asymptomatic even with significant volumes, their majority is already in advanced stage upon diagnosis [53]. Additionally, although modern ultrasonography can detect even tiny amounts of peritoneal fluid (down to 1 ml), there would be technical difficulties with its safe collection in very small volumes [56]. Therefore, early identification of asymptomatic ovarian cancer patients will be a challenge, if ascitic fluid spectroscopy is considered for clinical application in the future. It could, however, facilitate the detection of early-stage malignancy in cases with presumed benign ovarian pathology and some degree of ascites, as these patients do not routinely undergo a diagnostic biopsy.

In a broader perspective, clinical translation of spectroscopy faces some more general issues. For example, Raman equipment is robust, difficult to transfer and expensive. Additionally, many clinicians are unaware of spectroscopy’s potential in diagnosis and classification of disease. Development of user-friendly software programs and education of healthcare professionals could increase knowledge about spectroscopic techniques and gradually lead to their routine use in clinical practice [57].

A limitation of this study is the small size of its patient cohort. This is a common problem in research studies using human biological samples, which are often difficult to obtain and require complex consent forms for their acquisition. Additionally, ovarian cancer is an uncommon disease, making the recruitment of large patient cohorts challenging.

In conclusion, our study found that ascites, by means of predominantly collagen-related spectrochemical changes, achieved better classification between ovarian cancer and benign gynaecological conditions. However, its superiority to blood plasma and serum should be considered with caution, as other studies have shown better performances for these two biofluids. The obtained sensitivities, specificities and accuracies of around 80% with ascitic fluid Raman spectroscopy are satisfactory, though not high enough to allow for immediate translation into clinical practice. Future studies with larger cohorts, conducting regression analyses to eliminate possible confounding factors, could lead to optimised outcomes. The potential of ascitic fluid should be further investigated with other spectroscopic techniques, as well.
